# Notes and News

**Published:** 1901-02-16

**Authors:** 


### NOTES AND NEWS.

HOSPITAL MEETINGS.

Great Northern Central Hospital.—Annual meeting, Friday, February 22nd, 5 p.m.

One of the most recent contributions to the Prince of Wales's Hospital Fund is very touching. It comes from "The Jewish Residents in Durban, in Memory of Her late Majesty Queen Victoria."

The honorary secretaries of the Prince of Wales's Hospital Fund state that there is no intention of making any alteration whatever in the title of the Fund. Its full title is, it should be remembered, "The Prince of Wales's Hospital Fund for London to commemorate the Sixtieth Year of the Queen's Reign." At a recent meeting of the Fund an address was framed to present to the King.

The interesting report upon arsenic in beer made to the Local Government Board by Dr. Buchanan has now been published. His investigations establish beyond question the presence of a large proportion of arsenic in the sulphuric acid used in the preparation of glucose or brewery sugar. He expresses himself as satisfied by the steps taken by the trade to stop the circulation of harmful beer as soon as the mischief was discovered, and further he is able to report that the tests now adopted would condemn beer containing as small a quantity as one part of arsenic in 1,500,000 parts of beer.

The useful little hospital at Surbiton, known as The Gables, and maintained by Sir Alfred Cooper for the reception of patients from the Princess of Wales's hospital ship, has just finished its work. The hospital ship has no longer any patients to draft into the hospital. Since last March about 150 sick and wounded soldiers have been received. The institution excited much local interest. A number of the medical men of the locality rendered gratuitous medical service, and Sir Alfred Cooper's financial burden has been lightened by the kind co-operation of some of the tradespeople. Messrs. Packham & Sons provided bread for the establishment, and the West London Dairy Company furnished the milk. The undertaking has revealed proofs of much generosity, kindly feeling, and judicious management.

The annual meeting of the London Fever Hospital will be held at the Hotel Victoria on Friday, the 15th inst., at 5 p.m. Lord Balfour of Burleigh will preside.

We regret to announce that Mr. Frederic Andrew, for 46 years the valued secretary of the Royal Hospital for Incurables, died on the 22nd ult. after a few days' illness.

The Chairman of the National Canine Defence League, High Elms, Nutfield, Surrey, appeals to the charitable for funds to defray the cost of quarantine imposed upon the dogs of soldiers returning from South Africa. The cost of the keep of the dogs when in quarantine is from 5s. to 10s. per week.

Smallpox is still very prevalent in Paris. In Glasgow where there are still a large number of cases, comparative statistics are available, collected by Dr. R. Thomson, of the Glasgow Smallpox Hospital, showing the protective powers of vaccination in affording immunity from the worst forms of the disease.

A vigorous crusade against rats is being carried on at Cardiff, as it has been proved beyond doubt that some rats found dead had died of the plague. At Cape Town, where rats are also reported to have succumbed to the disease, a reward of 3d. per head has been offered for rats, and a special staff is engaged to exterminate them in the docks.

Those who favour the establishment of a separate hospital for Jews in Manchester are not inclined to abandon the scheme, and to stimulate interest in the scheme, the opportunity of rendering it a memorial to Queen Victoria has been taken advantage of. This may possibly be a sufficiently good pretext to induce the Jewish population to support a project which however does not appeal to their business instincts. We have before in this connection pointed out the Jewish ward at the London Hospital as an example of a satisfactory arrangement. It seems to us a pity that money is not put to the best use by securing a Jewish ward instead of a Jewish hospital. The accusation of extravagance would attend the scheme for a small hospital of only 20 beds in a city already provided with the necessary establishments in its hospitals to carry out a project at a much smaller cost.

Dr. Robertson, of Paisley, is trying to arouse local interest to secure a sanatorium for consumptives at Paisley. He has gone very fully into the scheme which he lays before the Paisley public. He is very much in favour of weather-proof iron or wooden buildings, as opposed to those of stone and lime, principally on account of the smaller cost. But in endeavouring to establish the advantages of the less permanent buildings, he rather overstates the necessary cost of stone and lime establishments. He quotes £300 per bed as a reasonable sum to expect to expend. But for the character of the buildings required for a sanatorium this sum is extraordinarily high, and we venture to think it must mislead to state that the choice lies between an expenditure of £300 and £80 per bed. When expert advice is obtained it will be found there are some very excellent alternatives between the two forms of structures he notes.

